# Fermented Wheat Germ Extract (Avemar) in the Treatment of Cardiac Remodeling and Metabolic Symptoms in Rats

**DOI:** 10.1093/ecam/nep090

**Published:** 2011-01-04

**Authors:** Abishek Iyer, Lindsay Brown

**Affiliations:** School of Biomedical Sciences, The University of Queensland, Brisbane, 4072, Australia

## Abstract

Avemar, a product of industrial fermentation of wheat germ with a standardized content of benzoquinone and plant flavonoids, has been tested as an anti-cancer and immunomodulatory dietary supplement. Proposed mechanisms include anti-oxidant and anti-inflammatory actions. This study has determined whether these actions of Avemar may also be useful in the treatment of cardiovascular diseases. Two experimental rat models of cardiovascular remodeling were used in this project: the deoxycorticosterone acetate (DOCA)-salt-induced model of chronic hypertension (study I) and a high-carbohydrate/high-fat diet-induced model producing chronic symptoms of the metabolic syndrome and its associated cardiovascular complications (study II). Our results in these rat models of hypertension and diet-induced obesity show that treatment with Avemar improved cardiac function, decreased macrophage infiltration resulting in decreased collagen deposition in the ventricular myocardium, reversed an increased stiffness of the left ventricle in the diseased hearts and attenuated increased plasma malondialdehyde concentrations. In addition to the changes in the heart, Avemar reversed glucose intolerance, normalized systolic blood pressure and decreased visceral fat deposition in rats fed a high-fat/high-carbohydrate diet. In conclusion, the fermented wheat germ extract Avemar has a potential role in attenuating chronic hypertension, diabetes or metabolic syndrome-induced cardiovascular symptoms along with metabolic abnormalities such as glucose tolerance and obesity.

## 1. Introduction

Avemar is a product of industrial fermentation of wheat germ with a standardized content of benzoquinone and plant flavonoids that has been reported as safe and effective as an anti-cancer and immunomodulatory dietary supplement [1–6]. Avemar benefited patients with stage III melanoma or colorectal cancer by increasing progression-free survival [1, 3]. Avemar supplementation reduced the incidence of treatment-related febrile neutropenia in children with solid cancers [7]. Avemar is currently being evaluated as a potential adjuvant therapeutic agent in human cancers, including those of the breast, colon, lung and prostate, with promising results as a supportive therapy with current anti-cancer therapies [3, 7–9]. Preliminary results with Avemar supplementation in severe rheumatoid arthritis patients showed improved quality of life after 6 and 12 months of treatment compared with baseline measurements [10, 11].

Plants have provided many possible treatment options for human hypertension and metabolic diseases [12–17]. As with this wheat germ extract, the ingredients of the plant extracts responsible for the therapeutic responses are usually not known. The mechanisms which could possibly explain the responses to wheat germ extract include inhibition of poly(ADP-ribose) polymerase (PARP), decrease in MHC class I molecules, up-regulation of intercellular adhesion molecule-1 (ICAM-1), regulation of pentose phosphate pathway, inhibition of cyclooxygenase isoforms and up-regulation of endogenous antioxidants [3, 6, 9, 18, 19]. These mechanisms may also be relevant for the treatment of cardiovascular and metabolic diseases. Decreased antioxidant concentrations resulting in oxidative stress may play an important role in the etiology of the symptoms of cardiovascular remodeling such as hypertension and hypertrophy; these antioxidant concentrations may be improved by nutraceuticals [20, 21]. Selective cyclooxygenase and PARP inhibitors as well as antioxidant compounds have improved cardiovascular function in myocardial ischemia/reperfusion injury, heart failure, cardiomyopathies, circulatory shock, cardiovascular aging, diabetic cardiovascular complications, myocardial hypertrophy, atherosclerosis and vascular remodeling following injury [22–24].

Since the progression of cardiac remodeling and metabolic diseases is characterized by oxidative stress and chronic inflammation, it is possible that Avemar decreases cardiovascular remodeling, glucose intolerance and fat deposition through its reported anti-oxidant and anti-inflammatory properties. Thus, we have investigated whether dietary treatment with Avemar can regulate cardiovascular remodeling and metabolic responses in hypertensive and diet-induced obese rats. Structural changes in the heart were characterized by histology and echocardiography, whereas heart function was measured *in vivo* using echocardiography and *ex vivo* in isolated perfused hearts. Isolated thoracic rings were used to measure vascular reactivity.

## 2. Methods

### 2.1. Male Wistar Rats

Male Wistar rats were bred at The University of Queensland Biological Resources facility. All experimental protocols were approved by the Animal Experimentation Ethics Committee of The University of Queensland, under the guidelines of the National Health and Medical Research Council of Australia. Rats were given *ad libitum* access to food and water and were housed in 12-h light/dark conditions. Body weight, food and water intakes were measured daily. Two experimental models of cardiovascular remodeling were used in this project: the deoxycorticosterone acetate (DOCA)-salt-induced model of chronic hypertension (study I) and a high-carbohydrate/high-fat diet-induced model producing chronic symptoms of the metabolic syndrome and its associated cardiovascular complications (study II).

All the rats in study I were uninephrectomized (UNX). Rats, 8-9 weeks old, were divided into four experimental groups of eight rats each; UNX only or treated with Avemar (4% mixture in powdered rat food; UNX + AVE) or UNX together with DOCA (25 mg in 0.4 mL dimethylformamide s.c. every fourth day) and 1% NaCl in the drinking water, with or without Avemar treatment (DOCA and DOCA + AVE groups). For uninephrectomy, rats were anesthetized with an intraperitoneal injection of tiletamine (25 mg/kg) and zolazepam (25 mg/kg, Zoletil) together with xylazine (10 mg/kg, Rompun); a lateral abdominal incision provided access to the kidney, and the left renal vessels and ureter were ligated. The left kidney was removed and weighed, and the incision site was sutured. Avemar treatment was started 4 days before surgery and continued for 28 days until the experiments were performed (treatment for 32 days).

Study II consisted of three experimental groups of 8-9 weeks old male Wistar rats treated for 16 weeks with corn starch (CS) (*n* = 12), cafeteria + beef tallow (CAF + BT) (*n* = 12) and cafeteria + beef tallow + Avemar (CAF + BT + AVE) (*n* = 12) protocol, respectively. The CAF + BT diet consisted of fructose (175 g), powdered rat food (155 g), beef tallow (200 g), condensed milk (395 g), Hubble, Mendel and Wakeman salt mixture (25 g) and water (50 mL) per kilogram of food. The drinking water in the CAF + BT-fed rats was augmented with 25% fructose in the water. For the control diet, fructose and condensed milk were replaced with CS (575 g) and BT was replaced with water (200 mL). Avemar was administered as a 4% mixture along with CAF + BT diet for 8 weeks starting 8 weeks after the initiation of the high-fat/high-carbohydrate diet (reversal protocol). Daily caloric intake was calculated from the food and water intakes using the following values (kJ/g): fructose, 15.4; CS, 15.9; condensed milk, 13.8; BT, 37.7 and rat food, 13.5.

Systolic blood pressure was measured in study I rats after 0, 2 and 4 weeks and in study II rats after 0, 4, 8, 12 and 16 weeks under light sedation with i.p. injection of Zoletil (tiletamine 15 mg/kg, zolazepam 15 mg/kg). Measurements were taken using an MLT1010 Piezo-Electric Pulse Transducer (ADInstruments, Sydney, Australia) and inflatable tail-cuff connected to a MLT844 Physiological Pressure Transducer (ADInstruments) and PowerLab data acquisition unit (ADInstruments).

Fasting blood glucose concentrations were measured for study II animals with blood taken from the tail vein using Medisense Precision Q.I.D glucose meter (Abbott Laboratories, Bedford, USA). The rats were given 40% glucose solution in distilled water (2 g/kg body weight) via oral gavage. Tail vein blood samples were taken at 0, 30, 60, 90 and 120 min following glucose administration.

Plasma malondialdehyde concentrations as a measure of oxidative stress were determined in post-mortem blood by HPLC [26].

### 2.2. Echocardiography

Echocardiography was performed by trained cardiac sonographers at the Small Animal Practice, School of Veterinary Sciences, The University of Queensland or The Prince Charles Hospital, Brisbane, small animal theater. Rats were anesthetized via i.p. injection with Zoletil (tiletamine 15 mg/kg, zolazepam 15 mg/kg) and Ilium Xylazil (xylazine 10 mg/kg). Echocardiographic images of rats were obtained using the Hewlett Packard Sonos 5500 (12 MHz frequency fetal transducer) at an image depth of 3 cm using two focal zones. Measurements of left ventricular posterior wall thickness and internal diameter were made using two-dimensional *M*-Mode taken at mid-papillary level [25].

### 2.3. Isolated Heart Preparation

The left ventricular function of the rats in all treatment groups was assessed using the Langendorff heart preparation. Terminal anesthesia was induced via i.p. injection of pentobarbitone sodium 100 mg/kg (Lethabarb). Once anesthesia was achieved, heparin (1000 IU) was injected into the right femoral vein. Isovolumetric ventricular function was measured by inserting a latex balloon catheter into the left ventricle connected to a Capto SP844 MLT844 physiological pressure transducer and Chart software on a Maclab system. All left ventricular end-diastolic pressure values were measured by pacing the heart at 250 beats per minute using an electrical stimulator. End-diastolic pressures were obtained starting from 0 mmHg up to 30 mmHg. The right and left ventricles were separated and weighed. Diastolic stiffness constant (*κ*, dimensionless) was calculated as in previous studies [26, 27].

### 2.4. Organ Bath Studies

Thoracic aortic rings (4 mm in length) were suspended in an organ bath chamber with a resting tension of 10 mN. Cumulative concentration—response (contraction) curves were measured for noradrenaline; concentration—response (relaxation) curves were measured for acetylcholine and sodium nitroprusside in the presence of a submaximal (70%) contraction to noradrenaline [28].

### 2.5. Organ Weights

Following euthanasia, the heart, liver, kidneys, visceral fat pads and spleen were removed and blotted dry for weighing. All organ weights except visceral fat pads were normalized relative to the body weight at the time of their removal (in mg/g). Visceral fat pads were normalized relative to tibial length at the time of removal (in mg/mm).

### 2.6. Histology

Collagen distribution was measured in the left ventricle following staining with picrosirius red and analyzed by laser confocal microscopy. Tissues were initially fixed for 3 days in Telly's fixative (100 mL of 70% ethanol, 5 mL of glacial acetic acid and 10 mL of 40% formaldehyde) and then transferred into modified Bouin's fluid (85 mL of saturated picric acid, 5 mL glacial acetic acid and 10 mL of 40% formaldehyde) for 2 days. The samples were then dehydrated and embedded in paraffin wax. Thick sections (15 *μ*m) were cut and stained with image analysis under the laser scanning microscope performed as previously described [26, 29]. Thin sections (10 *μ*m) of left ventricle were cut and stained with hematoxylin and eosin for determination of inflammatory cell infiltration.

### 2.7. Statistical Analysis

All data sets were represented as mean ± SEM. Comparisons or findings between groups were made via statistical analysis of data sets using one-way/two-way analysis of variance followed by the Duncan test to determine differences between treatment groups. A *P*-value < .05 was considered as statistically significant.

### 2.8. Drugs

DOCA, heparin, noradrenaline, acetylcholine and sodium nitroprusside were purchased from Sigma Chemical Company (St Louis, MO, USA.) The fermented wheat germ extract, Avemar, was provided by Jenny Blyth, Medimpex Pty Ltd, Mudgeeraba, QLD 4213, Australia and thoroughly mixed in the food to a final concentration of 4%. Noradrenaline, acetylcholine and sodium nitroprusside were dissolved in distilled water. DOCA was dissolved in dimethylformamide with mild heating. Fructose, CS and BT were obtained through The University of Queensland Chemical Store.

## 3. Results

### 3.1. DOCA-Salt-Induced Rat Model of Cardiac Remodeling (Study I)

Hypertension developed in DOCA-salt rats together with an increased water intake, but these rats failed to gain weight (Table 1). Avemar treatment did not change systolic blood pressure and body weight but attenuated the increased water intake (Table 1). Intake of Avemar, calculated from the daily food intake, was not significantly different between the treated groups: 2.4 ± 0.1 (UNX + AVE) and 2.3 ± 0.3 (DOCA + AVE) g/kg body wt/day, respectively. Plasma malondialdehyde concentrations, as a measure of oxidative stress, were increased in DOCA rats; this increase was attenuated by Avemar treatment (Table 1).

#### 3.1.1. Cardiac Structure and Function

Hearts from DOCA-salt rats showed marked cardiac hypertrophy, as evidenced by increased left ventricular wet weight relative to body weight and left ventricular mass derived from echocardiography (Table 1). This was associated with a decrease in left ventricular internal diameter, indicating concentric cardiac hypertrophy (Table 1). Avemar failed to alter the decrease in left ventricular chamber diameter (Table 1). Additionally, the relative wet weights of the liver, spleen and the remnant kidney from DOCA-salt rats were increased (Table 1). Treatment with Avemar failed to attenuate the increased wet weights of the ventricles and the remnant kidney. Increased wet weight of both liver and spleen were attenuated with Avemar treatment (Table 1).

Echocardiographic assessment of heart function showed that Avemar attenuated the increase in relative wall thickness observed in DOCA-salt rats (Table 1). Furthermore, aortic blood flow velocities were increased and cardiac output was decreased in DOCA-salt rats; Avemar prevented the increase in blood flow velocities but failed to improve cardiac output (Table 1).

**Figure 1:**
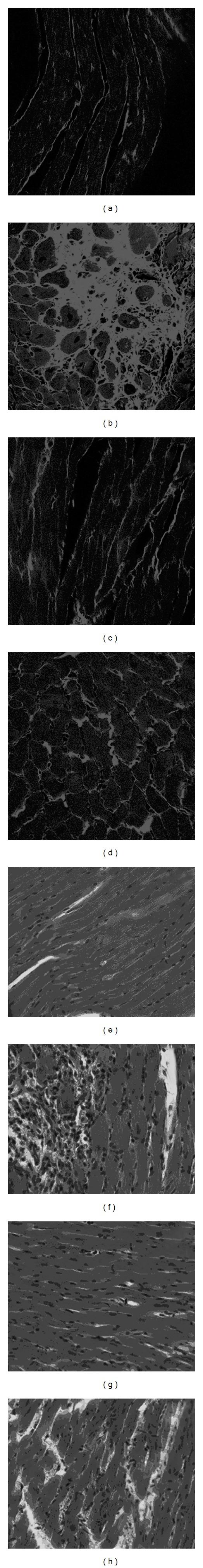
Picrosirius red staining of left ventricular interstitial collagen deposition (magnification, ×40) in UNX (a), DOCA (b), UNX + AVE (c), DOCA + AVE (d)-treated rats and hematoxylin and eosin staining of infiltrating inflammatory cells of left ventricular interstitial region (magnification, ×40) in UNX (e), DOCA (f), UNX + AVE (g), DOCA + AVE (h)-treated rats; collagen is stained light red.

Functionally, the increased diastolic stiffness in isolated hearts from DOCA-salt rats was prevented by Avemar treatment (Table 1). Furthermore, both interstitial and perivascular collagen deposition was increased in the left ventricle of DOCA-salt rats compared to the UNX rats; this deposition was markedly attenuated by Avemar treatment (Table 1 and Figure 1) Spatial location of monocyte/macrophages determined by hematoxylin and eosin (H&E) staining (Figure 1) showed monocyte/macrophages in the left ventricle of UNX rats in very low numbers and always as single cells. The density of monocyte/macrophages found in the left ventricle of DOCA-salt rats was significantly greater than in UNX, and these cells were usually found in clusters of cells located at scar sites and throughout the interstitium and the areas of fibrosis. Very few infiltrating cells were found in scar tissue in DOCA-salt rats treated with Avemar, predominantly due to the decreased area of scar tissue within the left ventricle; few infiltrating cells were found in the perivascular areas (Figure 1).

#### 3.1.2. Vascular Function

**Figure 2:**
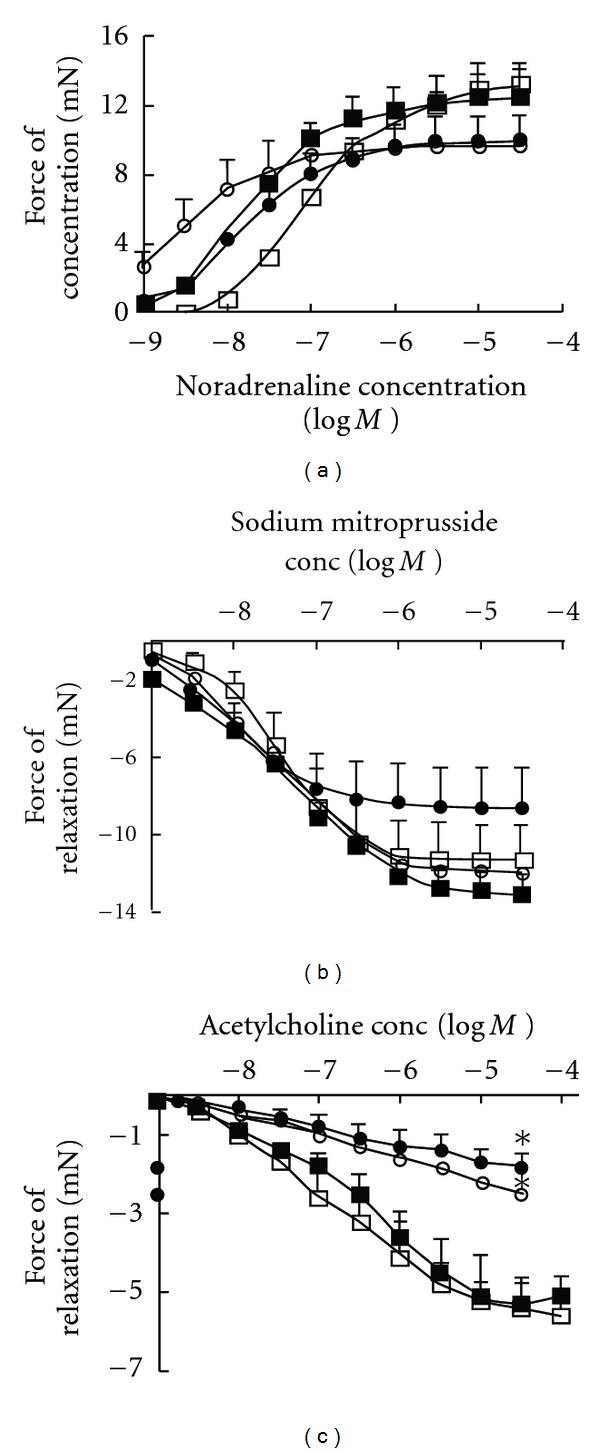
Cumulative concentration—response curves for noradrenaline (a), sodium nitroprusside (b) and acetylcholine (c) in thoracic aortic rings from UNX (filled square), UNX + AVE (open square), DOCA (filled circle) and DOCA + AVE (open circle)-fed rats after 16 weeks of feeding. *P < .05 versus UNX.

In isolated thoracic aortic rings, DOCA-salt rats showed decreased contractile responses to noradrenaline (Figure 2(a)), unaltered responses to sodium nitroprusside (Figure 2(b)) together with endothelial dysfunction, defined as decreased relaxation responses to acetylcholine (Figures 2(c)). Treatment with Avemar failed to normalize these decreased responses (Figures 2(a) and 2(c)).

### 3.2. High-Carbohydrate/High-Fat Diet-Induced Model of Cardiac Remodeling and Metabolic Changes (Study II)

Young male Wistar rats fed a high-carbohydrate/high-fat diet showed increased body weight, especially with increased abdominal fat pads, compared with CS-fed rats, without an increased food intake (Table 2). Caloric intake was similar in both diets with CS-fed rats averaging 408 ± 38 kJ/day, whereas CAF + BT-fed rats averaged 381 ± 47 kJ/day. Treatment with Avemar reversed the increase in body weight and abdominal fat pads compared to the CAF + BT-fed rats. Measurement of blood glucose concentrations after administration of a loading dose of glucose showed that CAF + BT-fed rats were glucose-intolerant as the blood glucose concentrations reduced much more slowly than in the corn starch-fed rats (Table 2). Treatment with Avemar normalized the glucose tolerance compared with CAF + BT-fed rats (Table 2). 

#### 3.2.1. Cardiac Structure and Function

**Figure 3:**
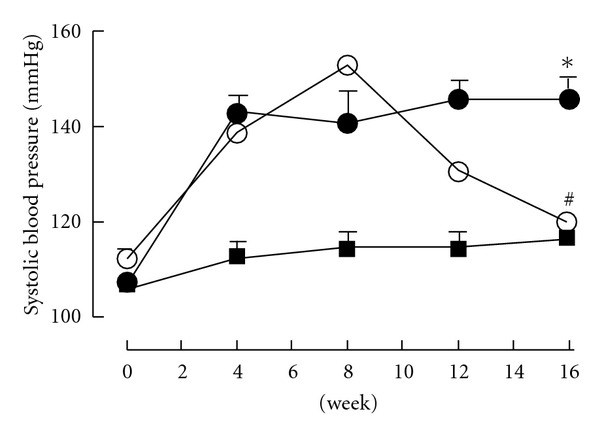
Tail-cuff measurement of systolic blood pressure recorded at 0, 4, 8, 12 and 16 weeks for corn starch (filled square), CAF + BT (filled circle) and CAF + BT + AVE (open circle)-fed rats. *P< .05 versus corn starch-fed rats; ^#^ *P*< .05 versus CAF + BT-fed rats.

Systolic blood pressure increased in CAF + BT-fed rats over the first 4 weeks and was then maintained over the next 12 weeks with mean values of 141 ± 6 mmHg at 8 weeks and 146 ± 4 mmHg at 16 weeks. In contrast, the systolic blood pressure in corn starch-fed rats was unchanged at 115 ± 3 mmHg at 8 weeks and 117 ± 4 mmHg at 16 weeks (Figure 3). Initiating treatment with Avemar after 8 weeks of CAF + BT feeding decreased blood pressure to 131 ± 1 mmHg at 12 weeks and 120 ± 2 mmHg at 16 weeks (Figure 3). Echocardiographic assessment of CAF + BT-fed rats *in vivo* indicated ventricular dilatation (increased internal diameter in diastole) with increased ventricular mass and left ventricular systolic volume but with decreased contractility shown as a decreased fractional shortening and ejection fraction (Table 2). CAF + BT-fed rats showed decreased mitral valve flow rates (evaluated as a ratio of the flow rate (E/A)) compared with the corn starch-fed rats (Table 2). *Ex vivo* cardiac function as measured in the Langendorff isolated heart showed a markedly increased cardiac stiffness (Table 2). These changes in cardiac structure and function were attenuated by Avemar treatment in the CAF + BT-fed rats (Table 2). 

#### 3.2.2. Organ Remodeling and Oxidative Stress

**Figure 4:**
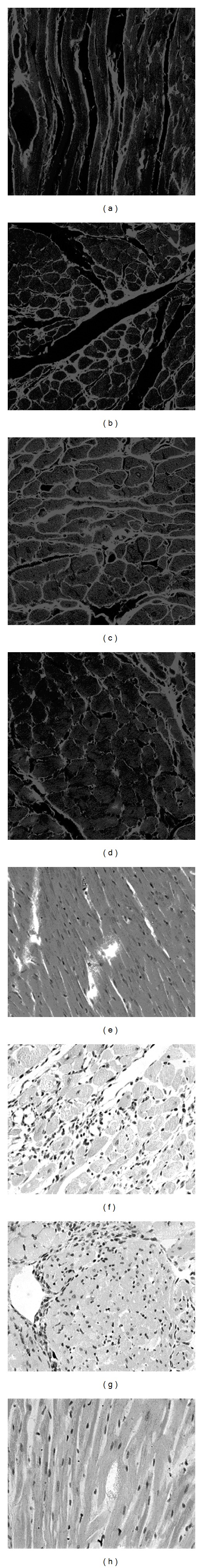
Picrosirius red staining of left ventricular interstitial collagen deposition in corn starch (16 weeks) (a), CAF + BT (8 weeks) (b), CAF + BT (16 weeks) (c) and CAF + BT + AVE (16 weeks) (d)-treated rats and hematoxylin and eosin staining of infiltrating inflammatory cells of left ventricular interstitial region (magnification, ×40) CS (16 weeks) (e), CAF + BT (8 weeks) (f), CAF + BT (16 weeks) (g) and CAF + BT + AVE (16 weeks)-treated (h) rats; collagen is stained light red.

Post-mortem organ weights showed a selective increase in left ventricular wet weight and kidney weights compared with the right ventricle and other major organs in CAF + BT-fed rats compared with corn starch-fed rats (Table 2). Avemar administration reversed this organ hypertrophy in CAF + BT-fed rats. Spatial location of monocyte/macrophages determined by H&E staining (Figure 4) shows more monocyte/macrophages in the left ventricle of the CAF + BT rats than in corn starch-fed rats, and these cells were usually found in clusters of cells in the interstitial and perivascular regions of fibrosis (Table 2 and Figure 4). Furthermore, both interstitial and perivascular collagen deposition was markedly increased in the left ventricle of CAF + BT-fed rats compared to the corn starch-fed rats (Table 2 and Figure 4). Plasma malondialdehyde concentrations, as a marker of oxidative stress, were increased with CAF + BT feeding. Avemar treatment attenuated these increases in ventricular collagen deposition and plasma malondialdehyde concentrations (Table 2).

#### 3.2.3. Vascular Responses

**Figure 5:**
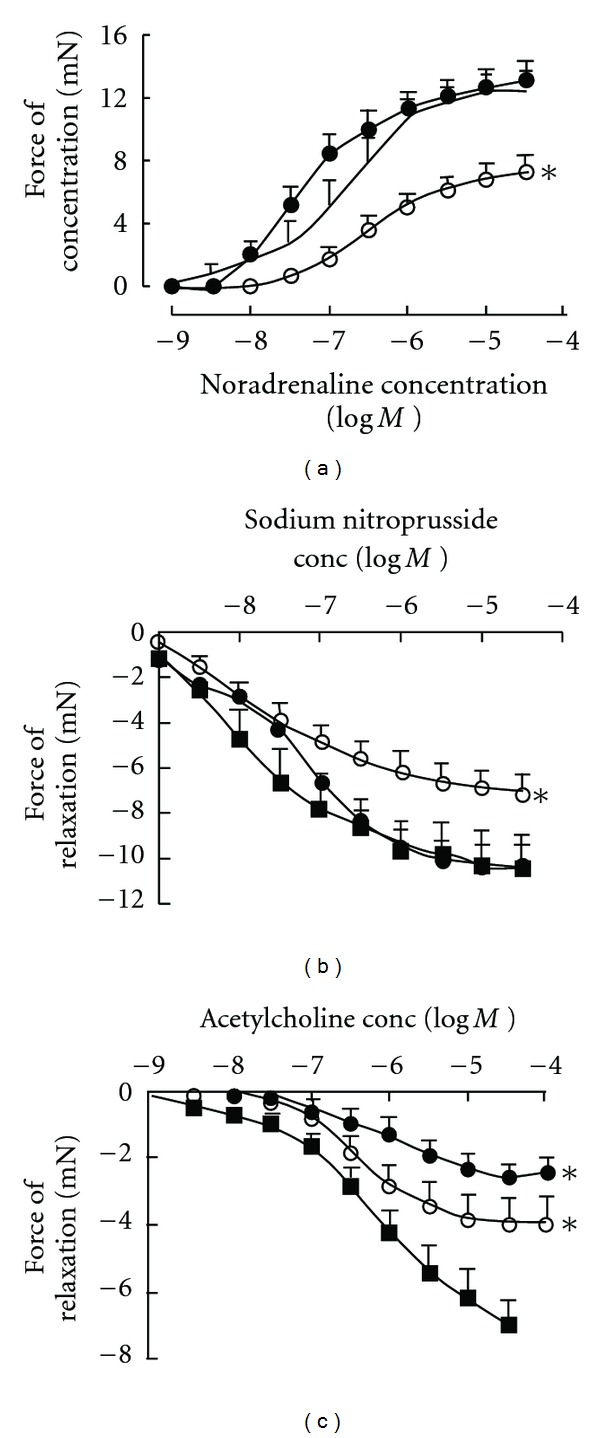
Cumulative concentration-response curves for noradrenaline (a), sodium nitroprusside (b), and acetylcholine (c) in thoracic aortic rings from corn starch (filled square), CAF + BT (filled circle) and CAF + BT + AVE (open circle)-fed rats after 16 weeks of feeding. *P < .05 versus corn starch-fed rats

Vascular responses to noradrenaline were unchanged by CAF + BT feeding. Responses to sodium nitroprusside (endothelium-independent relaxation) were decreased by CAF + BT feeding (Figures 5(a) and 5(b)). CAF + BT feeding induced endothelial dysfunction, shown as decreased responses to the endothelium-dependent relaxant, acetylcholine (Figure 5(c)). Treatment with Avemar failed to normalize this decreased response to acetylcholine but normalized the decreased response to sodium nitroprusside (Figures 5(b) and 5(c)). Furthermore, Avemar-treated rats showed decreased responses to noradrenaline compared with corn starch and CAF + BT-fed rats (Figure 5(a)).

## 4. Discussion

In the DOCA-salt rat model of hypertension, we have shown that treatment with Avemar improved cardiac function, decreased macrophage infiltration resulting in decreased collagen deposition in the ventricular myocardium, reversed an increased stiffness of the left ventricle in the diseased hearts and attenuated oxidative stress measured as plasma malondialdehyde concentrations without changing systolic blood pressure. Our previous studies have shown similar results with inhibitors of the renin-angiotensin system (captopril, candesartan and spironolactone) [27]. In contrast, we showed that treatment of DOCA-salt rats with L-arginine also decreased blood pressure in the DOCA-salt hypertensive rat [19]. In the current study, we have further shown that Avemar reversed glucose intolerance, normalized blood pressure and decreased visceral fat deposition in rats fed a high-fat/high-carbohydrate diet.

**Figure 6:**
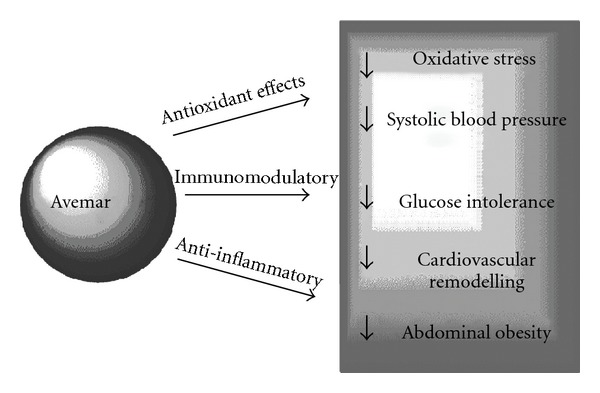
Potential mechanisms for the beneficial effects of Avemar in diet-induced obese rats.

Since the mechanisms for these cardiovascular actions may be an extension of the anti-oxidant, anti-inflammatory and immunomodulatory mechanisms proposed for the anti-cancer actions of Avemar, it is worthwhile considering whether these actions improve cardiovascular function in disease models (Figure 6). Avemar produced non-specific inhibition of both cyclooxygenase isoforms, with IC_50_ values on COX-1 and COX-2 of 100 *μ*g/ml and 300 *μ*g/ml, respectively [30]. In animal studies, COX inhibition decreased myocardial infarct size [24], produced scar thinning [31, 32], attenuated cardiopulmonary dysfunction during endotoxemia [31, 32] and prevented angiotensin II-induced production of superoxide in cardiovascular tissue, together with the decrease in systolic blood pressure and cardiac hypertrophy *in vivo* [33]. Arachidonic acid metabolites are important mediators of inflammation in cardiovascular remodeling; arachidonic acid is released from membranes by phospholipase A2. Inhibition of phospholipase A2 with KH064 prevented an increase in infiltration of inflammatory cells, myocardial collagen deposition and ventricular stiffness in young spontaneously hypertensive rats [29]. Thus, the decreased collagen deposition following Avemar treatment could be mediated by these known actions as inhibitors of the production of arachidonic acid metabolites. Further, Avemar decreased inflammation through other immunomodulatory mechanisms, such as decreased production of pro-inflammatory cytokines IL-6 and IL-10 in mice with systemic lupus erythematosus [20, 34] and decreased macrophage infiltration in animal models of rheumatoid arthritis [20, 34]. Thus, the proposed mechanisms for the anti-cancer effects of Avemar may contribute to the cardiovascular responses in this study.

Furthermore, Avemar contains compounds such as benzoquinones and other plant flavonoids, important agents in controlling oxidative stress and cell damage [19, 34]. There is extensive literature on the therapeutic use of antioxidant compounds to improve cardiovascular function [20, 21]. Epidemiological comparisons between populations and studies within populations also support the contention that high plasma concentrations or increased dietary intake of natural antioxidant vitamins may protect against the development of cardiovascular diseases in humans [20]. Thus, an improvement in oxidative status following Avemar treatment may improve cardiovascular function.

Since inflammatory mediators are important in fat deposition [35], the anti-inflammatory mechanisms underlying the anticancer effects of Avemar may also decrease abdominal fat deposition. Furthermore, peroxisome proliferator-activated receptor *δ* (PPAR*δ*) is of central importance in fat oxidation [35–37]. Flavonoids and phenolic acids decreased intracellular triglyceride and glucose phosphate dehydrogenase activity through down-regulating expression of adipogenic transcription factors such a PPAR*δ* and up-regulating adiponectin expression [38]. The decrease in fat deposition and weight gain in the rats fed with a high-carbohydrate/high-fat diet with Avemar treatment could be explained by the actions of flavonoids possibly modulating adipogenic transcription factors.

Similarly, the anti-inflammatory mechanisms useful in treatment of cancer may contribute to the changes in glucose tolerance in the high-carbohydrate/high fat diet rats treated with Avemar [39, 40]. Antioxidants such as N-acetylcysteine, vitamin C and E have also shown anti-diabetic effects possibly by protecting pancreatic *β*-cells from glucose toxicity, stimulating insulin secretion and moderately reducing blood glucose levels [41–43]. One of the consequences of elevated blood glucose concentrations is the non-enzymatic glycation of proteins such as hemoglobin A1c (HbA1c) and serum albumin [44]. Plant flavonoids inhibited fructose-mediated glycation of albumin improving the symptoms of diabetes [44].

Other components of Avemar such as the benzoquinones may also be cardioprotective. Benzoquinones have very similar characteristics to vitamins and are anti-oxidant compounds. Coenzyme Q10 (ubiquinone) is a naturally occurring benzoquinone, which may prevent cellular damage during myocardial ischemia and reperfusion by its roles in oxidative phosphorylation and membrane stabilization [45]; diabetes induced a decrease in coenzyme Q plasma levels [46]. Coenzyme Q10 and alpha-tocopherol treatment decreased glycated HbA1c and pancreatic lipid peroxidation in diabetic rats [40]. Coenzyme Q10 has also been used in oral form to treat various cardiovascular disorders including angina pectoris, hypertension and congestive heart failure [45, 47].

In conclusion, our results show that the fermented wheat germ extract Avemar has a potential role to attenuate the cardiovascular symptoms induced by hypertension, diabetes or the metabolic syndrome while moderating metabolic abnormalities such as glucose tolerance and obesity. Since Avemar is already available in humans as a complementary therapy for cancer, this product could be further evaluated in a clinical setting as an adjunct therapy for preventing cardiovascular and metabolic symptoms.

## Figures and Tables

**Table 1:** Physiological parameters of UNX, UNX + AVE, DOCA and DOCA + AVE-fed rats.

Parameter	UNX (4 weeks)	UNX + AVE (4 weeks)	DOCA (4 weeks)	DOCA + AVE (4 weeks)
Daily water intake (mL)	67 ± 11 (*n* = 6)	59 ± 9 (*n* = 6)	190 ± 33* (*n* = 6)	125 ± 30** (*n* = 6)
Daily food intake (g)	29 ± 2 (*n* = 6)	27 ± 3 (*n* = 6)	24 ± 5 (*n* = 6)	23 ± 5 (*n* = 6)
Systolic blood pressure (mmHg) 0 week	107 ± 1.3 (*n* = 6)	113 ± 1.8 (*n* = 6)	108 ± 3.1 (*n* = 6)	105 ± 2.1 (*n* = 6)
Systolic blood pressure (mmHg) 2 weeks	128 ± 0.7 (*n* = 6)	145 ± 6* (*n* = 6)	176 ± 7.0* (*n* = 6)	171 ± 6.0* (*n* = 6)
Systolic blood pressure (mmHg) 4 weeks	135 ± 1.7 (*n* = 6)	151 ± 1.0* (*n* = 6)	186 ± 3.6* (*n* = 6)	182 ± 3.0* (*n* = 6)
LVIDd (cm)	0.66 ± 0.01 (*n* = 6)	0.57 ± 0.01* (*n* = 6)	0.48 ± 0.02* (*n* = 6)	0.51 ± 0.01* (*n* = 6)
LVPWd (cm)	0.15 ± 0.01 (*n* = 6)	0.17 ± 0.01 (*n* = 6)	0.22 ± 0.01* (*n* = 6)	0.22 ± 0.01* (*n* = 6)
Fractional shortening (%)	54 ± 6 (*n* = 6)	57 ± 1.4 (*n* = 6)	58 ± 3 (*n* = 6)	64 ± 5 (*n* = 6)
LV mass (g)	0.7 ± 0.01 (*n* = 6)	0.63 ± 0.01 (*n* = 6)	0.85 ± 0.01* (*n* = 6)	0.75 ± 0.01** (*n* = 6)
Cardiac output (mL/min)	56 ± 9 (*n* = 6)	39 ± 8 (*n* = 6)	21 ± 3* (*n* = 6)	27 ± 8* (*n* = 6)
Ejection fraction (%)	87 ± 3 (*n* = 6)	92 ± 0.7 (*n* = 6)	92 ± 2 (*n* = 6)	92 ± 1.4 (*n* = 6)
Relative wall thickness	0.49 ± 0.01 (*n* = 6)	0.94 ± 0.2* (*n* = 6)	1.49 ± 0.68* (*n* = 6)	0.94 ± 0.2** (*n* = 6)
LV—interstitial collagen (% of total area)	2.7 ± 0.3 (*n* = 5)	2.4 ± 0.5 (*n* = 5)	11.7 ± 1.3* (*n* = 6)	3.8 ± 0.7** (*n* = 5)
LV—perivascular collagen (% of total area)	25.6 ± 0.9 (*n* = 4)	26.2 ± 1.1 (*n* = 4)	36.4 ± 1.3* (*n* = 4)	28.1 ± 1.0** (*n* = 4)
Diastolic stiffness constant (*κ*)	20.3 ± 0.8 (*n* = 6)	19.7 ± 1.8 (*n* = 6)	32.3 ± 1.7* (*n* = 6)	22.6 ± 0.7** (*n* = 6)
LV + septum (mg/g body wt)	2.16 ± 0.1 (*n* = 6)	2.0 ± 0.1 (*n* = 6)	3.06 ± 0.08* (*n* = 6)	2.9 ± 0.1* (*n* = 6)
RV (mg/g body wt)	0.36 ± 0.01 (*n* = 6)	0.51 ± 0.01* (*n* = 6)	0.49 ± 0.01* (*n* = 6)	0.54 ± 0.01* (*n* = 6)
Liver (mg/g body wt)	28.2 ± 1.7 (*n* = 6)	40.6 ± 1.4 (*n* = 6)	54 ± 2.0* (*n* = 6)	45.6 ± 3** (*n* = 6)
Spleen (mg/g body wt)	2.4 ± 0.17 (*n* = 6)	2.4 ± 0.1 (*n* = 6)	4.3 ± 0.2* (*n* = 6)	3.1 ± 0.1** (*n* = 6)
Remnant kidney (mg/g body wt)	5.1 ± 0.4 (*n* = 6)	4.0 ± 0.5 (*n* = 6)	8.2 ± 0.3* (*n* = 6)	9.3 ± 0.5* (*n* = 6)
Plasma malondialdehyde (MDA) concentration (*μ*mol/L)	19.7 ± 0.7 (*n* = 6)	20.4 ± 0.6 (*n* = 6)	27.3 ± 0.8* (*n* = 6)	23.9 ± 0.6** (*n* = 6)

Values are mean ± SEM; number of experiments in parentheses. LV, left ventricle; RV, right ventricle; LVIDd, left ventricular internal diameter at diastole; LVPWd, left ventricular posterior wall thickness at diastole. LV mass calculated according to [25]. *P < .05 versus UNX; **P < .05 versus DOCA.

**Table 2:** Physiological parameters of CS, CAF+BT and CAF+BT+AVE-fed rats.

Parameter	Corn starch (16 weeks)	CAF + BT (8 weeks)	CAF + BT (16 weeks)	CAF + BT + AVE (16 weeks)
Body weight (g)	341 ± 13 (*n* = 8)	448 ± 11 (*n* = 8)	523 ± 14 (*n* = 8)	479 ± 8 (*n* = 8)
Daily water intake (mL)	24.0 ± 2.4 (*n* = 6)	19.1 ± 3.0 (*n* = 6)	21.6 ± 4.0 (*n* = 6)	19.0 ± 3 (*n* = 6)
Daily food intake (g)	29.0 ± 4.3 (*n* = 6)	26.1 ± 2.8 (*n* = 6)	22.4 ± 1.8 (*n* = 6)	22.0 ± 4 (*n* = 6)
Daily drug intake (g/kg/day)	N/A	N/A	N/A	1.83 ± 0.25 (*n* = 6)
Fasting plasma glucose concentrations (mmol/L)	3.2 ± 0.5 (*n* = 6)	3.5 ± 0.1 (*n* = 6)	4.0 ± 0.1* (*n* = 6)	3.6 ± 0.1** (*n* = 6)
Plasma glucose concentration (mmol/L) (after 120 min glucose loading)	6.0 ± 0.4 (*n* = 4)	5.9 ± 0.2 (*n* = 6)	7.0 ± 0.4* (*n* = 6)	4.4 ± 0.2** (*n* = 6)
LVIDd (cm)	0.61 ± 0.02 (*n* = 6)	0.78 ± 0.01* (*n* = 8)	0.76 ± 0.02* (*n* = 8)	0.73 ± 0.01* (*n* = 8)
LVPWd (cm)	0.18 ± 0.01 (*n* = 4)	0.16 ± 0.01 (*n* = 8)	0.20 ± 0.01 (*n* = 8)	0.17 ± 0.01 (*n* = 8)
Fractional shortening (%)	63 ± 2 (*n* = 4)	44 ± 2* (*n* = 8)	35 ± 1* (*n* = 8)	54 ± 2** (*n* = 8)
Mitral valve flow rate (E/A) ratio	2.1 ± 0.1 (*n* = 6)	1.8 ± 0.1 (*n* = 6)	1.1 ± 0.01* (*n* = 6)	1.4 ± 0.12** (*n* = 6)
Estimated LV mass (g)	0.82 ± 0.06 (*n* = 4)	0.88 ± 0.03 (*n* = 8)	1.01 ± 0.05* (*n* = 8)	0.82 ± 0.03** (*n* = 8)
LV systolic volume (mL)	0.046 ± 0.01 (*n* = 5)	0.09 ± 0.01* (*n* = 8)	0.127 ± 0.01* (*n* = 8)	0.040 ± 0.01** (*n* = 8)
Cardiac output (mL/min)	93 ± 6 (*n* = 4)	98 ± 7 (*n* = 8)	83 ± 9 (*n* = 8)	105 ± 6 (*n* = 8)
Ejection fraction (%)	95 ± 0.9 (*n* = 4)	81 ± 2* (*n* = 8)	72 ± 1* (*n* = 8)	90 ± 0.9** (*n* = 8)
Relative wall thickness	0.55 ± 0.04 (*n* = 4)	0.41 ± 0.01* (*n* = 8)	0.51 ± 0.04 (*n* = 8)	0.45 ± 0.01** (*n* = 8)
LV—interstitial collagen (% of total area)	4.8 ± 0.5 (*n* = 4)	14.6 ± 1.4* (*n* = 4)	19.9 ± 1.2* (*n* = 4)	8.4 ± 0.4** (*n* = 4)
LV—perivascular collagen (% of total area)	21.2 ± 1.6 (*n* = 4)	31.2 ± 2.6* (*n* = 4)	35.3 ± 3.0* (*n* = 4)	26.1 ± 1.2** (*n* = 4)
Diastolic stiffness constant (*κ*)	20.5 ± 0.4 (*n* = 4)	26.3 ± 2.2* (*n* = 6)	27.8 ± 1.5* (*n* = 6)	21.6 ± 1.4** (*n* = 6)
LV + septum (mg/g body wt)	1.9 ± 0.2 (*n* = 4)	2.1 ± 0.03 (*n* = 8)	2.2 ± 0.07 (*n* = 8)	1.7 ± 0.1** (*n* = 8)
RV (mg/g body wt)	0.53 ± 0.03 (*n* = 4)	0.41 ± 0.01* (*n* = 8)	0.43 ± 0.02* (*n* = 8)	0.44 ± 0.02* (*n* = 8)
Liver (mg/g body wt)	26.7 ± 0.6 (*n* = 4)	28.2 ± 1.4 (*n* = 8)	29.9 ± 0.95 (*n* = 8)	23.1 ± 0.02** (*n* = 6)
Spleen (mg/g body wt)	2 ± 0.1 (*n* = 4)	1.8 ± 0.03 (*n* = 8)	1.68 ± 0.1 (*n* = 8)	1.75 ± 0.23 (*n* = 8)
Left and right kidney (mg/g body wt)	5.15 ± 0.1 (*n* = 4)	6.3 ± 0.2* (*n* = 8)	5.94 ± 0.12* (*n* = 8)	5.13 ± 0.39** (*n* = 8)
Abdominal fat pads (mg/mm tibial length)	401 ± 56 (*n* = 6)	343 ± 25 (*n* = 8)	826 ± 62* (*n* = 8)	339 ± 31** (*n* = 8)
Plasma malondialdehyde (MDA) concentration (*μ*mol/L)	26.9 ± 0.7 (*n* = 5)	29.4 ± 0.5* (*n* = 5)	32.2 ± 1.2* (*n* = 5)	28.8 ± 0.6** (*n* = 5)

Values are mean ± SEM; number of experiments in parentheses. LV, left ventricle; RV, right ventricle; LVIDd, left ventricular internal diameter at diastole; LVPWd, left ventricular posterior wall thickness at diastole. LV mass calculated according to [25]. *P <  .05 versus CS; **P <  .05 versus CAF + BT.
